# Parliament and the Profession

**Published:** 1916-01-01

**Authors:** 


### PARLIAMENT AND THE PROFESSION.

### Questions in the House of Commons.

QUESTIONS in the House of Commons relating to matters of medical interest have not been so numerous, and there has been a falling-off in the number of interrogations concerning National Health Insurance administration, possibly because the questioners have exhausted almost every conceivable matter suggesting controversy without getting the satisfaction they desired. In spite of Mr. Tennant's reassuring statements with regard to the provision made for the dental treatment of soldiers, there is a growing feeling that the provision made is altogether inadequate. The employment of unqualified "dentists" is a scandal, and casts a grave reflection on the Army authorities and all in any way responsible for so great a mistake.

### Alleged Shortage of Army Dentists.

SIR C. KINLOCH-COOKE asked a series of questions with regard to the arrangements that had been made for the treatment of the teeth of soldiers on service abroad and at home. He alleged that a number of officers and men were suffering from a complaint of the jaw which could have been obviated had there been a sufficient number of dentists on the spot, that there were eight Red Cross hospitals in France without a dentist, and that men invalided home from the Front for teeth had often to remain in this country a much longer time than would otherwise be necessary owing to the insufficient dental arrangements at the various hospitals. To all these questions Mr. Tennant gave the customary reassuring replies. He asserted that a considerable number of full-time dental officers had already been appointed to the troops abroad, and that any more that might be asked for would be sent. There was no evidence of any increased number of soldiers being invalided for defective teeth in proportion to the numbers of men in the field. With regard to the dental treatment of troops in England, he said that in each command a number of full-time commissioned dental officers had been appointed, and that the general officers commanding were also empowered to employ as many civilian dentists as there was need for.

### Other Matters of Interest.

MEDICAL STUDENTS AS SURGICAL DRESSERS.—Replying to Mr. Pringle, Mr. Tennant stated that medical students are not being employed in military hospitals as surgical dressers and medical clerks.

ROYAL ARMY MEDICAL CORPS.—The strength of Regular officers in the R.A.M.C. is 1,084 and that of temporary officers 5,891. This is exclusive of the Special Reserves and Territorial Force.

THE INSURANCE COMMISSIONERS AND SCOTTISH CHEMISTS.—Mr. C. Roberts asked whether, before refusing the offer of Scottish panel chemists to continue during 1916 the arrangements for Insurance dispensing on the existing terms, he had accepted the advice of the Scottish Insurance Commissioners, refused to state whether or not he had accepted the advice, and would only say that he had received it. Subsequently Mr. Roberts announced that terms had been arranged with the Scottish chemists, but he did not state what the terms were. The chemists, however, have won their point, and will continue service under conditions much more favourable than those which it was attempted to force on them.

